# Removal of Congo Red and Methylene Blue from Aqueous Solutions by Vermicompost-Derived Biochars

**DOI:** 10.1371/journal.pone.0154562

**Published:** 2016-05-04

**Authors:** Gang Yang, Lin Wu, Qiming Xian, Fei Shen, Jun Wu, Yanzong Zhang

**Affiliations:** 1 State Key Laboratory of Pollution Control and Resource Reuse, School of the Environment, Nanjing University, Nanjing, China; 2 Institute of Ecological and Environmental Sciences, Sichuan Agricultural University, Chengdu, China; NERC Centre for Ecology & Hydrology, UNITED KINGDOM

## Abstract

Biochars, produced by pyrolyzing vermicompost at 300, 500, and 700°C were characterized and their ability to adsorb the dyes Congo red (CR) and Methylene blue (MB) in an aqueous solution was investigated. The physical and chemical properties of biochars varied significantly based on the pyrolysis temperatures. Analysis of the data revealed that the aromaticity, polarity, specific surface area, pH, and ash content of the biochars increased gradually with the increase in pyrolysis temperature, while the cation exchange capacity, and carbon, hydrogen, nitrogen and oxygen contents decreased. The adsorption kinetics of CR and MB were described by pseudo-second-order kinetic models. Both of Langmuir and Temkin model could be employed to describe the adsorption behaviors of CR and MB by these biochars. The biochars generated at higher pyrolysis temperature displayed higher CR adsorption capacities and lower MB adsorption capacities than those compared with the biochars generated at lower pyrolysis temperatures. The biochar generated at the higher pyrolytic temperature displayed the higher ability to adsorb CR owing to its promoted aromaticity, and the cation exchange is the key factor that positively affects adsorption of MB.

## Introduction

Earthworms play an important role in the environmental ecology. They are involved in the decomposition of organic matter and in nutrient cycling [1]. Currently, earthworm farms are widely promoted for ecologically treating sludge, poultry feces, toxic organic waste, etc [2]. Vermicompost, the worm castings generated by the degradation of organic waste by earthworm [3], is characterized by a homogenous texture, well-developed porous structure, and large specific surface area. In addition, vermicompost also contains abundant mineral elements, humus, microbes, and enzymes [4]. Therefore, vermicompost is widely used in farms to promote plant growth, increase crop yield, improve soil environment, and condense heavy metals [5]. However, the organic waste (feed) can contain heavy metals, organic pollutants, and microbial germs resulting in the vermicompost being contaminated with the incompletely degraded harmful substances [6]. As a result, the traditional practice of directly applying vermicompost can be potentially detrimental for the environment.

Previous studies have shown that pyrolysis can solidify the heavy metal in biomass (especially in poultry manure and municipal sludge), reduce the content of organic pollutants and deactivate microorganisms [7, 8]. Biochar, the solid by-product of pyrolysis, has an abundance of pores and surface functional groups, along with a large specific surface area, high surface negative charge, and high charge density [9, 10]. These features allow biochar to be used as an adsorbent for pollutants [11, 12]. Thus, pyrolysis essentially reduces pollutants and generates environmentally friendly products. The applications of biochar (such as in the adsorption of pollutants) is primarily limited by its physical and chemical properties [13]. The pyrolysis temperature is the key factor that regulates the physical and chemical properties of the resulting biochar [8]. Biochars derived at a higher pyrolysis temperature display higher porosity and possess larger specific surface area that can promote nonspecific adsorption. Meanwhile, biochar derived at a lower pyrolysis temperature displays an abundance of surface functional groups and higher organic content that can improve specific adsorption. Further analysis of the influence of pyrolysis temperature on vermicompost-derived biochar can guide future efforts towards the design of suitable biochars for specific adsorption application.

Dyes, widely used in textile dyeing, paper printing, and other industries, can eventually accumulate in streams and other water sources. Owing to their toxic, mutagenic or carcinogenic properties, the presence of these pollutants in water can cause serious public concerns with regards to human health and growth of aquatic fauna and flora [14]. Thus, more researchers are investing their efforts towards the development methods for the treatment of dye-contaminated waste water. An adsorption-based approach, on account of its simple design and inexpensive nature, can be very effective in treating dye-contaminated waste-water [15]. Recently, biochars have been used as inexpensive adsorbents for use in dye-contaminated waste-water treatment facilities. However, the efficiency of the treatment has been found to be dependent on the protocol (predominantly, the pyrolysis temperature) employed to prepare the biochar. In this study, our objective was to investigate the influence of pyrolysis temperature on the properties of biochars and their potential to adsorb dyes. The results from this study can guide the efforts to develop an energy-saving protocol for the generation of highly efficient adsorbent.

## Materials and Methods

### Materials

In this work, no specific permissions were required for the locations or activities because no field studies were involved. Of course, it can be confirmed that no endangered or protected species were involved here. The vermicompost was collected from an earthworm farm in Chengdu, China. The earthworms were bred on cow manure. The collected vermicompost was dried at 80°C for 24 hours to remove moisture and then powdered. Then resulting powder (filtered through a 100 mesh sieve) was stored for subsequent use. Analytical-grade samples of congo red (CR), methylene blue (MB), NaOH, and HCl were procured from commercial sources.

### Preparation of biochars

Dried vermicompost (100g) was placed in a horizontal quartz vessel into the tubular furnace under N_2_ with flow rate of 0.03L·min^−1^. The furnace was programmed to initially increase the temperature at the rate of 10°C·min^−1^ to reach the predetermined temperature and then held at the final temperature for 2.0 h. 3 different final temperatures were set at 300°C, 500°C, and 700°C, respectively. After the completion of the pyrolysis, the biochar in the reactor was allowed to cool down to room temperature by turning off the furnace. The biochars derived after heating the vermicompost at 300°C, 500°C, and 700°C were named VM300, VM500, and VM700, respectively.

### Characterization of biochars

The elemental compositions of biochars were determined by dynamic flash combustion using a Vario MICRO elemental analyzer. Oxygen content was determined by calculating the difference between the measured mass and the mass accounted for carbon, hydrogen, and nitrogen. The pH of the biochar was measured in the supernatant of the aqueous solution of biochar (solid-water ratio was 1:20). Pore structure of biochars was characterized by nitrogen adsorption at 77K with automated surface area and pore size analyzer. The specific surface area was determined from the adsorption isotherms using the BET equation. The FT-IR spectra of the biochars were recorded on a Perkin Elmer Spectrum Two spectrophotometer. The cation exchange capacity (CEC) of the biochars was measured using sodium acetate for exchange, and determining the Na^+^ content using a flame spectrophotometer. The point of zero charge of biochar pH (pH_ZPC_) was determined based on a protocol reported previously [16]. The morphology of the biochars was observed on an S4800 scanning electron microscope (SEM).

### Batch adsorption experiment

Stock solutions of CR and MB (1000mg·L^−1^) were prepared by dissolving the appropriate amounts of the dyes in deionized water (1000mL). Batch adsorption experiments were conducted in 40mL Erlenmeyer flasks by incubating biochar (0.030g) with different concentrations of CR and MB in a solution (20mL). These flasks were shaken on a Roller Drum shaking incubator at a shaking speed of 8 rpm. Each of these experiments was performed in triplicate. Kinetic experiments were performed at 25°C. Solutions of the dyes (30mg·L^−1^) at pH 7.0 were incubated with biochar and the concentrations of CR and MB retained in supernatant solutions after different time intervals (0–360 min) were determined using an UV-vis spectrophotometer. The adsorption capacity of CR and MB were calculated according to the following equations:

$$q_{e}=({\text{C}}_{0}-C_{e})V/m$$ (1)

$$q_{t}=(C_{0}-C_{t})V/m$$ (2)

Where m(g) represents the mass of biochars and V (mL) is the volume of CR or MB solution. C_0_ and C_t_ (mg·L^−1^) are the initial and final (post-adsorption) concentrations of the dye solutions, respectively.

The effect of pH was evaluated by mixing biochar (0.030g) in a solution (20mL) of CR or MB (30mg·L^−1^) at 25°C. After 120 min of contact time, the concentrations of CR or MB retained in the supernatants were determined, and the equilibrium pH was recoded to investigate the relationship of pH and adsorption capacity. The effect of biochar dosage was studied at the initial pH of 7.0. Other conditions for this experiment were identical to that of evaluating the pH. To determine the adsorption isotherm, 0.030g biochar were added to independently to solutions of the dye (20mL) at different concentrations (5–200mg·L^−1^) in 40mL Erlenmeyer flasks at 25°C and monitored until the system reached adsorption equilibrium.

## Results and Discussion

### Characterization of biochars

The physicochemical properties of the three biochars are shown in Table 1. It is evident that the biochar yields from 91.56% to 71.81% as the pyrolysis temperature increased from 300°C to 700°C, and the corresponding ash content increases from 68.00% to 86.23%. These values are higher than those measure for other manure feedstock biochars generated at the same pyrolysis temperature due to the higher ash content of vermicompost itself (45.66%) [17–19]. During pyrolysis, the volatile components, which forms a large fraction of the surface functional group elements (C, H, N, and O) [7], are gradually lost. As a result, the C, H, N, and O content of the biochar decrease with increase in the pyrolysis temperature. The H/C molar ratio can be used as a parameter of carbonization. A low H/C ratio indicates high aromaticity [20]. The H/C molar ratio decreases with increasing pyrolysis temperature indicating that the biochar produced at higher pyrolysis has higher aromatic content and is more carbonaceous (Table 1). The O/C and (O+N)/C molar ratios are indicators for the polarity and surface oxygen functional groups of the biochar [21]. Low values of O/C and (O+N)/C ratio indicate low polarity and fewer oxygen-based functional groups. The O/C and (O+N)/C ratios decrease with the increase in pyrolysis temperature, suggesting a loss of oxygen-based functional groups at higher pyrolysis temperature with the simultaneous reduction in the polarity of biochar. In addition, the O/C ratio displays a positive correlation with CEC [22], indicating that the lower pyrolysis temperature-derived biochar has a higher CEC than that corresponding biochar obtained at higher pyrolysis. The CECs of the various biochars are the following: 159.81 mmol·kg^−1^ (VM300) > 112.82 mmol·kg^−1^ (VM500) > 104.52 mmol·kg^−1^ (VM700). Among the biochar samples, the lowest pH value (7.37) is observed for the biochar derived at lowest pyrolysis temperature (300°C). However, this value increases sharply and reaches 9.03 and 11.31 for the biochars derived at 500°C and 700°C, respectively. These increases in pH values can be attributed to the presence of a higher number of alkaline groups retained at higher pyrolysis temperature and the separation of alkali salts (K, Na, Ca, and Mg) from organic compounds [23].

**Table 1:** Physical and chemical properties of the biochars.

Biochars	Yield (%)	Ash content (%)	Elemental analysis				Atomic ratio			pH	pH_zpc_	CEC (mmol·kg^−1^)
			C(%)	H(%)	N(%)	O(%)	H/C	O/C	(O+N)/C			
VM300	91.56	68.00	19.10	2.39	1.67	8.84	0.13	0.46	0.55	7.37	8.13	159.81
VM500	73.88	81.07	13.52	0.88	0.98	3.55	0.07	0.26	0.34	9.03	8.52	112.82
VM700	71.81	86.23	12.46	0.42	0.58	0.31	0.03	0.02	0.07	11.31	8.78	104.52

Table 2 presents the pore structure parameters of the biochars. The BET surface area and total pore volume increase gradually with increase in the temperature, indicating an increase in the extent of raw material cracking and gradual development of the pore structure. In addition, the average pore diameters of the three biochars gradually decrease with the increase in the pyrolysis temperature, which indicates that the micropore structure is more readily formed at higher temperatures. The SEM images of these biochars are shown in S1 Fig. Biochar of VM300 surface consists of pores with large diameters due to the rapid volatilization of organic components in the vermicompost, and this is consistent with the lower specific area and large average pore size observed for VM300. When the pyrolysis temperature is 500°C, the generated biochar (VM500) has a surface pore structure which appears to have undergone additional melting, ablation, and appears to be a layered stack. Consequently, the specific surface area is enlarged. With further increase in the pyrolysis temperature, the lamellar structure of biochar (VM700) becomes thinner, smoother, and results in increased surface area, while the layered stack makes the pores smaller.

**Table 2:** Microstructure properties of the biochars.

Biochars	BET surface area (m^2^·g^−1^)	Total pore volume (mL·g^−1^)	Average pore diameter (nm)
VM300	24.332	0.09157	15.0528
VM500	46.224	0.1656	13.9146
VM700	76.296	0.1897	9.94406

In addition to physical/porous structure of the biochars, the adsorption capacity of biochars is also influenced by the nature of the functional groups on their surface [24]. S2 Fig presents the FT-IR spectra of the vermicompost chars produced at 3 different pyrolysis temperatures. The broad band at ~3435cm^−1^ can be attributed to O–H (phenolic, alcoholic, and carboxylic groups) or N–H (amino and amide groups) stretching. Adsorption bands at 2927cm^−1^ and 2855cm^−1^ indicate the presence of aliphatic C–H groups [25], while the band at 1634cm^−1^ represents the C = O stretching for aliphatic carboxyl and ketone groups [26]. The bands at 1515cm^−1^, 1451cm^−1^ and 1421cm^−1^ largely account for the amino and carboxyl groups. The presence of other functional groups/compounds, such as carbonyl (1385cm^−1^), cellulose/hemicellulose/lignin (1034cm^−1^indicates symmetric C–O stretching) and aromatic/heterocyclic compounds (800–500cm^−1^indicating C–H and C–N stretching) are also indicated [27]. With the increasing pyrolysis temperature, the adsorption intensities of the band at 3435cm^−1^ decrease, indicating a decrease in the number of–OH groups. Additionally, the intensities of the adsorptions in the 1634–1385cm^−1^ range and at ~1034cm^−1^ become weaker, indicating a decrease in the number of oxygen-containing functional groups. This is consistent with the lower O/C ratio observed for biochars generated at higher pyrolysis temperatures (see Table 1). The adsorption intensities at 2927cm^−1^ and 2855cm^−1^ disappear when the temperature is increased to 500°C. Moreover, the decrease in the intensities of the adsorption bands at 800–500cm^−1^ indicates a decrease in aliphatic hydrocarbon content and increase in the aromatic content in the biochars [7].

### Effect of adsorbent mass

**Fig 1:**
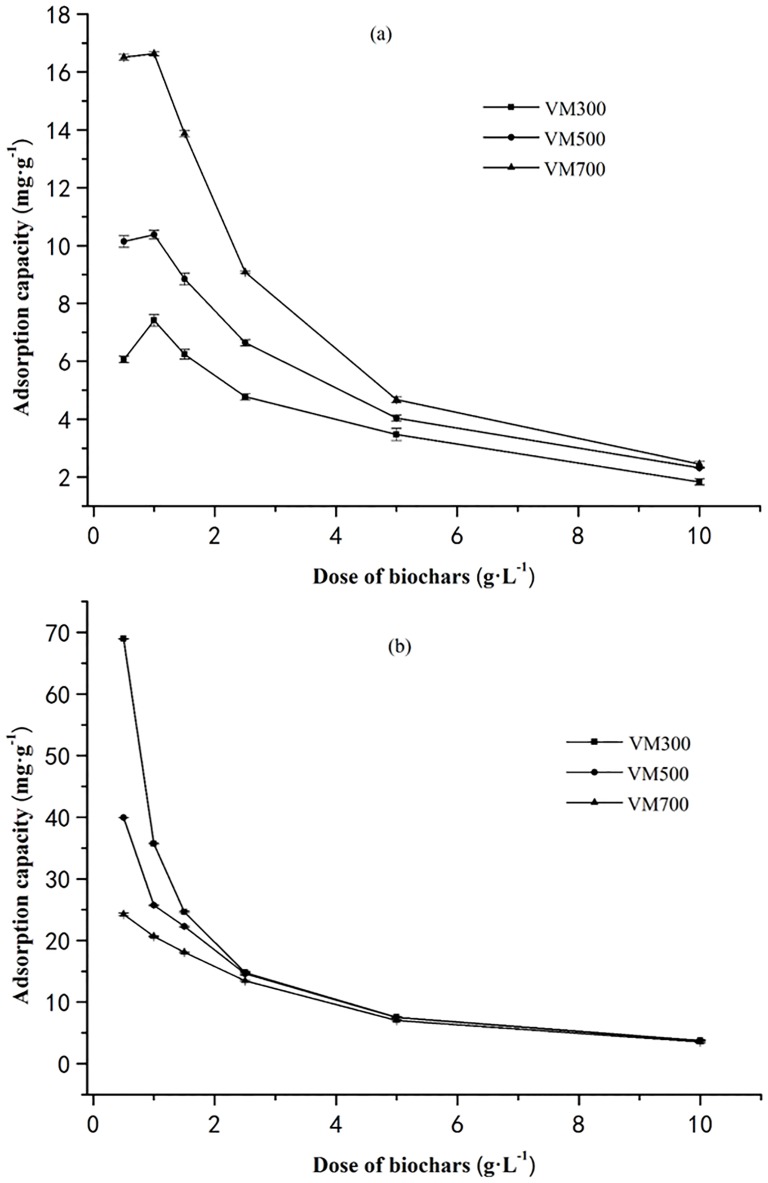
Effect of biochar dosages on the adsorption of CR (a) and MB (b).

Fig 1 shows the plot between adsorption capacities (mg·g^−1^) of the CR and MB against the dosage of the adsorbent (g·L^−1^). The adsorption capacity of CR was significantly affected by biochar dosage (p<0.05) when the dosage is lower than 5.0 g·L^−1^. However, the CR adsorption using the biochar obtained at different pyrolytic temperature could not be distinguished significantly (p<0.05) as the biochar dosage is higher than 5.0 g·L^−1^. As for MB, it also could be found that significant effects on the MB adsorption exists as biochar dosage is lower than 2.5 g·L^−1^. However, the adsorption capacity could not change significantly (p<0.05) as the dosage is higher than 2.5 g·L^−1^, even though the pyrolytic temperature is promoted. Overall, it is evident that the adsorption capacities of both CR and MB decrease with an increase in the adsorbent dosage of biochars in the 0.5–1.5 g·L^−1^ range. This is predominantly due to the splitting effect of flux (concentration gradient) between adsorbates and biochar, and the decrease in amount of dye adsorbed onto per weight of biochar happened consequently [28]. Another plausible reason for the decrease in the adsorption capacity with increasing dose of the adsorbent can be attributed to particle interactions, for example, aggregation. The aggregation can lead to a decrease in the total surface area of the adsorbent and increase in diffusional path length [29].

### Effect of pH

pH is a critical parameter influencing the uptake of dyes, since a change in pH can lead to ionization of the dye molecules or functional groups on the adsorbent [30]. The initial of CR and MB was adjusted to 3.0, 5.0 7.0 9.0 and 11.0, which covered wide range of pH. However, when the adsorption was in equilibrium, the corresponding pH of CR and MB solution was in alkalinity, moreover, the employed biochar with the higher temperature caused the higher alkalinity in CR and MB solution. These results were mainly attributed to the characteristics of high alkalinity and great buffering capacity of the employed biochars [31].

**Fig 2:**
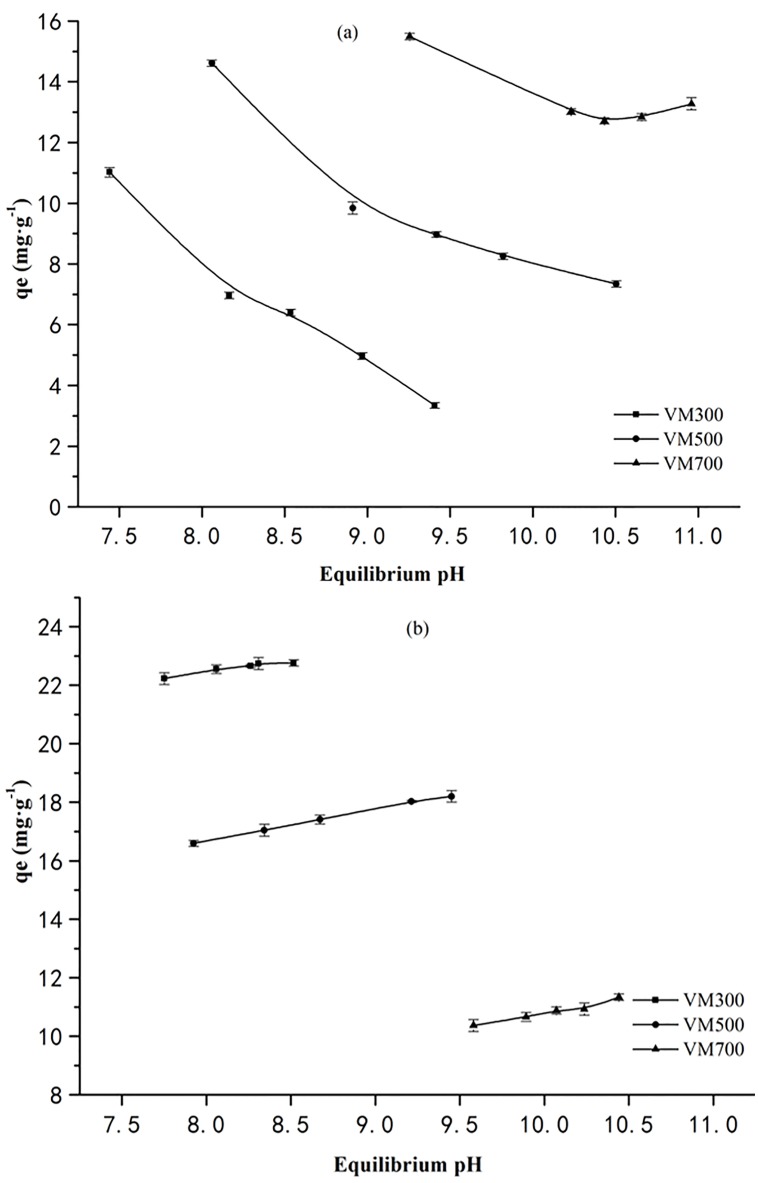
Relationships of equilibrium pH and the adsorption of CR (a) and MB (b) by biochars.

The relationship between equilibrium pH and adsorption of CR on vermicompost-derived biochars are shown in Fig 2a. Overall, the equilibrium pH of CR solution with biochar obtained from different temperature was ranged in 7.441–10.960. The negative correlation of adsorption capacities of CR and the equilibrium pH can be observed. Especially, the effects of equilibrium pH on CR uptake by VM300 and VM500 are significant (p<0.05). The pH_zpc_ of VM300, VM500, and VM700 were 8.17, 8.52, and 8.79, respectively. It is well known that electrostatic attraction exists between the negative charge of the anionic dye and the protonated—OH and—COOH groups on the surface of the biochars as equilibrium pH was lower than pH_zpc_ [32]. Therefore, the lower pH below the pH_zpc_ will facilitate adsorption. When the equilibrium pH was higher than pH_zpc_, the functional groups of the biochars are completely deprotonated and the electrostatic attraction will be weakened with pH increase, resulting in the decrease of CR adsorption. In addition, the OH^−^ions in the solution compete with the anionic CR molecules for the adsorption sites, leading to a decrease in adsorption of the anionic dye in higher pH, especially in alkalinity. Similar observations have been reported by Vimonses *et al*. *(2009)* during their investigations on kaolins as adsorbent for removing CR [32]. However, it was surprising that the CR adsorption capacities can be greatly improved by promoting the pyrolysis temperature for biochar, although the high alkalinity will weaken the adsorption. This result indicates there are some other potential actions to mainly control the CR adsorption by the biochar.

The relationship between equilibrium pH and adsorption of MB on vermicompost-derived biochars are shown in Fig 2b. Overall, the equilibrium pH of MB solution with biochar obtained from different temperature was ranged in 7.751–10.442. It could be obviously found that the effects of pH on the adsorption capacity of MB were not significant (p<0.05), although the positive correlation between equilibrium pH and MB adsorption was observed. These results are consistent with those reported previously that a high pH value facilitates the adsorption of MB using vegetal fiber activated carbons [33]. When pH values were lower than the pH_zpc_, the repulsive forces between positively charged functional groups on the surface of the adsorbent and the positively charged MB are significant. Furthermore, biochar releases a large fraction of Ca^+^, Mg^2+^, and K^+^ that compete with MB to bind to the adsorption sites. Therefore, these factors are not beneficial for the adsorption of MB. When the pH increase (pH>pH_zpc_), the surface of the adsorbent becomes negatively charged and the release of Ca^+^, Mg^2+^, and K^+^ are subdued, resulting in an increase in the adsorption of MB due to an increase in the electrostatic attraction between negatively charged adsorbent surface and positively charged dye [34]. Additionally, the biochar exhibited the stronger alkalinity as the biochar was produced at the higher temperature. Correspondingly, MB adsorption by the biochar was weakened by the higher pyrolysis temperature, implying that the cation exchange may be another important mechanism for MB uptake because the release of Ca^2+^, Mg^2+^ was restricted by higher OH^-^.

### Adsorption isotherm

The adsorption isotherm indicates how the adsorbate molecules are distributed between the liquid and solid phases when the adsorption process is in the equilibrium state [35]. Analysis of the fitness between the data and different isotherm models is an important step in determining the suitable model. 3 well-known models, including Langmuir, Freundlich and Temkin isotherms, are selected to rationalize the dye-biochar interactions observed in this study. Their linear forms of these 3 isotherm equations are expressed as:

$$C_{e}/{\text{q}}_{e}=1/q_{0}K_{L}+1/{\text{q}}_{0}C_{e}$$ (3)

$${\text{lnq}}_{e}=\text{ln}K_{F}+1/\text{nln}{\text{C}}_{e}$$ (4)

$$q_{e}=B\text{ln}A+B{\text{lnC}}_{e}$$ (5)

Where K_L_ (L·mg^−1^) and q_o_ (mg·g^−1^) represent Langmuir constant and the maximum adsorption capacity calculated by Langmuir model, respectively. K_F_ ((mg·g^−1^) (L·mg^−1^)^1/n^) and 1/n are Freundlich constants that describe the adsorption density and intensity, respectively. A and B are the Temkin constant.

**Fig 3:**
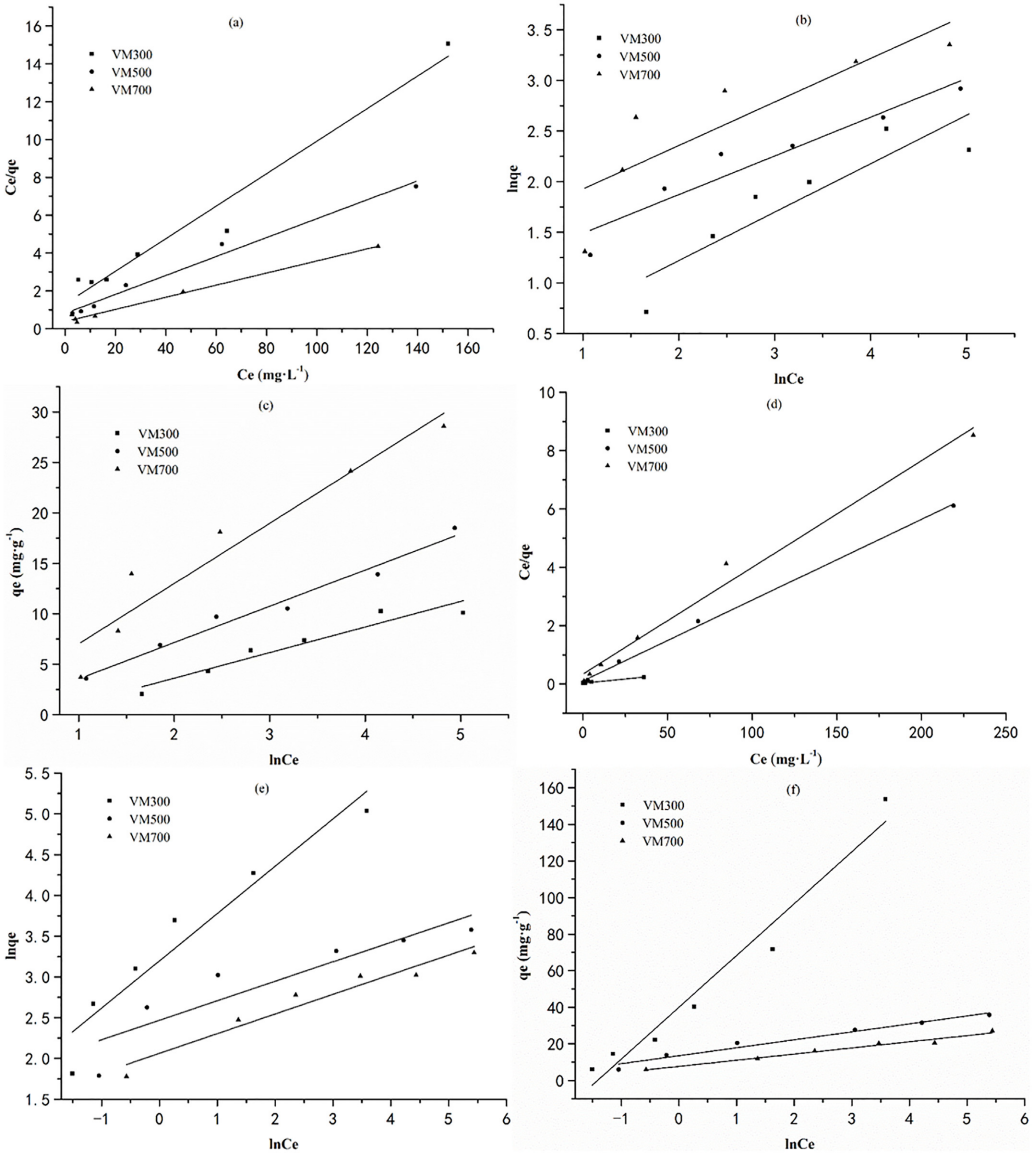
Adsorption isotherms for the retention of dyes on biochars. (a) Langmuir adsorption isotherm for adsorption of CR on biochars; (b) Freundlich adsorption isotherm for adsorption of CR on biochars; (c) Temkin adsorption isotherm for adsorption of CR on biochars; (d) Langmuir adsorption isotherm for adsorption of MB on biochars; (e) Freundlich adsorption isotherm for adsorption of MB on biochars; (f) Temkin adsorption isotherm for adsorption of MB on biochars.

The linear forms of the Langmuir, Freundlich and Temkin isotherms are shown in Fig 3 and the relevant constants are shown in Table 3. It could be easily found that the adsorption of MB and CR can be described by the model of Langmuir and Temkin according to the higher correlation coefficient (R^2^). Based on the result of good fitness of Langmuir, it could be deduced that a homogeneous surface containing equivalent sites on biochars, and they were available for CR and MB adsorption [36]. Moreover, the good fitness of Temkin isotherm indicated that the CR and MB adsorption can be mainly controlled by electrostatic interaction of chemical adsorption [37].

**Table 3:** Isotherm constants for the retention of dyes by biochars.

Dyes	Biochars	Langmuir constants			Freundlich constants			Temkin constants		
		q_m_(mg·g^−1^)	K_L_(L·mg^−1^)	R^2^	K_F_(L·mg^−1^)	N	R^2^	A	B	R^2^
CR	VM300	11.63	0.0653	0.9582	1.30	2.09	0.7668	0.564	2.535	0.908
	VM500	20.00	0.0614	0.9809	2.45	2.61	0.8967	0.987	3.599	0.970
	VM700	31.28	0.0832	0.9863	4.47	2.33	0.6656	1.191	5.981	0.907
MB	VM300	174.22	0.1962	0.9888	24.49	1.73	0.9073	4.110	28.322	0.954
	VM500	36.11	0.2557	0.9979	11.81	4.18	0.7935	22.427	4.351	0.963
	VM700	27.35	0.1061	0.9848	7.87	4.15	0.9319	9.933	3.358	0.968

The important characteristic of Langmuir isotherm, called dimensionless separation factor (R_L_) [38], is defined as:

$$R_{L}=1/(1+bC_{0})$$ (6)

Where C_0_ (mg·g^−1^) is the initial dye concentration and b (L·mg^−1^) is Langmuir constant. The value of R_L_ indicates the type of the isotherm as irreversible (R_L_ = 0), favorable (0<R_L_<1), linear (R_L_ = 1), and unfavorable (R_L_>1). The R_L_ for the adsorption of CR and MB onto biochars are in the range of 0.06687–0.4722 and 0.08357–0.5126, respectively. These values indicate that adsorption of CR and MB by biochars are favorable processes.

### Kinetic studies

**Fig 4:**
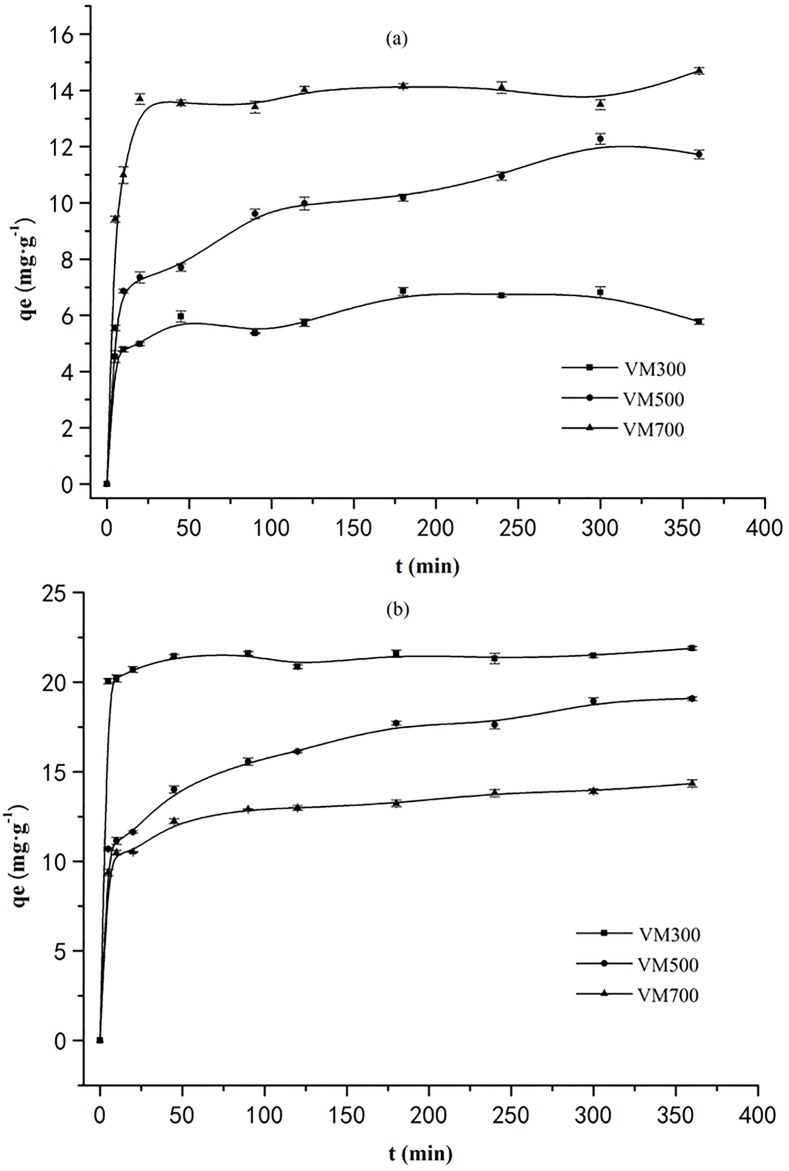
Time dependent adsorption of CR (a) and MB (b) by biochars.

The effects of contact time on the dyes adsorption by biochars are shown in Fig 4. Initially, the dyes are rapidly adsorbed. Subsequently, the adsorption increases slowly over time, until equilibrium is reached at 180, 300, and 120min for the adsorption of CR on VM300, VM500, and VM700, respectively. Similarly, the equilibrium can be achieved at 48, 300, and 90min for the adsorption of MB on VM300, VM500, and VM700, respectively. The pseudo-first-order and pseudo-second order kinetics, represented below, are often used to evaluate the kinetics and mechanism of solid-liquid adsorption [39].

$$\text{ln}(q_{e}-q_{t})=\text{ln}q_{e}-k_{1}t$$ (7)

$$t/q_{t}=1/k_{2}q_{e}^{2}+t/q_{e}$$ (8)

Where q_e_ and q_t_ refer to the adsorption capacity at equilibrium and at time t (min); k_1_ (min^−1^) and k_2_ (g·mg^−1^·min^−1^) are the adsorption rate constants for the pseudo-first-order and pseudo-second-order adsorptions.

The data analysis shows that the correlation coefficients (R^2^) for fitting the data to the pseudo-second-order kinetic model are higher (>0.9817) than those observed for fitting the data to the pseudo-first-order kinetic model (see Table 4). Additionally, in contrast to the values of q_e_ calculated using the pseudo-first-order, the q_e_ values determined using the pseudo-second-order kinetic model are more in agreement with the experimentally determined values (q_exp_). Thus, the pseudo-second-order kinetic model reasonably describes the mechanism of the CR and MB adsorption by the biochars. This observation supports the assumption that the adsorption is predominantly due to chemisorption [40] and is consistent with the adsorption equilibrium data being well represented by the Temkin isotherm equation.

**Table 4:** Fitted pseudo first-order and second-order kinetic models for the adsorption of dyes by biochars.

Dyes	Biochars	q_exp_(mg·g^−1^)	Pseudo-first order			Pseudo-second order		
			q_e_,cal(mg·g^−1^)	k_1_(min^−1^)	R^2^	q_e_,cal(mg·g^−1^)	k_2_(mg·g^−1^·min^−1^)	R^2^
CR	VM300	6.90	3.10	0.017	0.6443	6.34	0.051	0.9817
	VM500	11.23	4.97	0.010	0.9182	12.20	0.005	0.9905
	VM700	14.2	2.66	0.022	0.7708	14.32	0.021	0.9970
MB	VM300	21.80	0.95	0.0045	0.2338	21.71	0.033	0.9996
	VM500	19.08	9.74	0.0115	0.8778	19.44	0.0035	0.9958
	VM700	14.30	3.90	0.0082	0.9423	14.35	0.009	0.9985

Weber and Morris have proposed the intraparticle diffusion model to identify diffusion mechanisms in the adsorption process. The effect of intraparticle diffusion resistance on adsorption can be determined using the following equation [33]:

$$q_{t}=k_{t}^{1/2}+c$$ (9)

Where k_i_ (mg·g^−1^·min^–1/2^) is the intraparticle diffusion rate constant.

**Fig 5:**
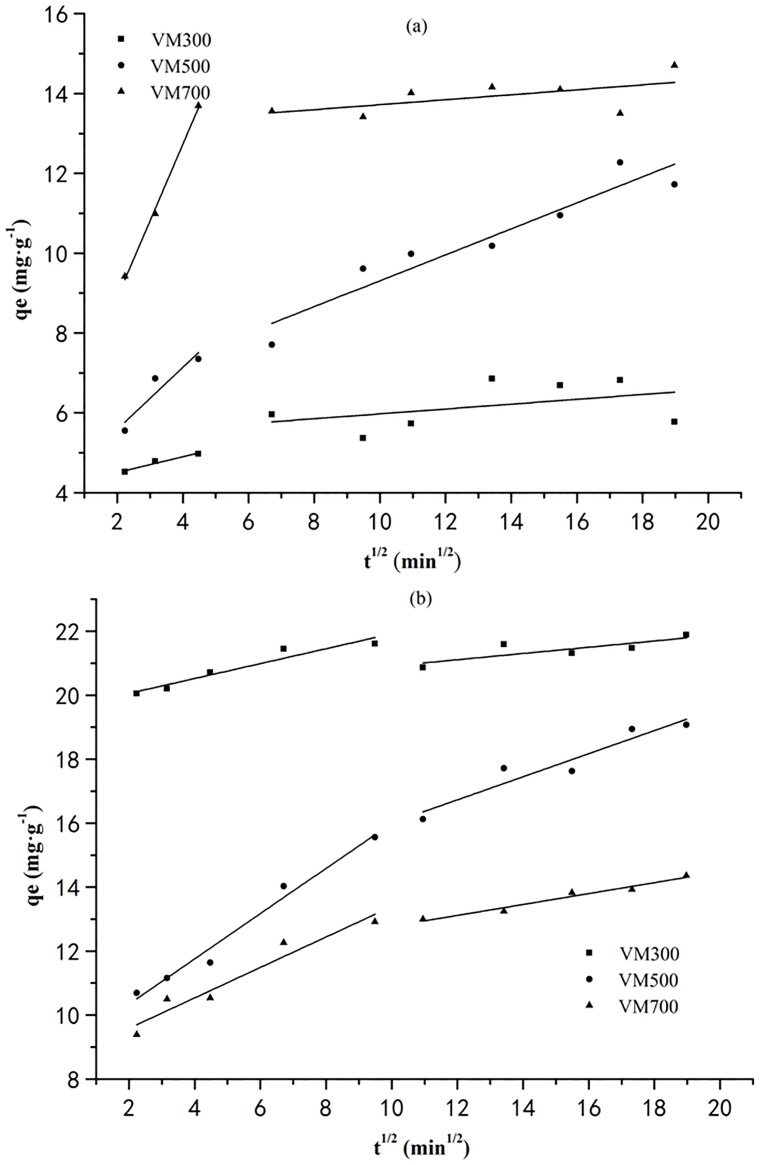
Intraparticle diffusion kinetic plots for the adsorption of CR (a) and MB (b) by biochars.

Fig 5 shows 2 regions of q_t_ versus t^1/2^ for the adsorption of dyes by biochars. The first region (2–5 and 2–10min^1/2^ range for CR and MB, respectively) has been interpreted to represent the stage of film diffusion, which is the diffusion of dye molecules in external surface of biochars. However, the second region (7–19 and 11–19min^1/2^range for CR and MB, respectively) represents the intraparticle diffusion stage, which is the diffusion of these dyes into the pores of biochars. Constants calculated for both regions are given in Table 5. The k_i1_ are obviously larger than k_i2_, indicating the intrapaticle diffusion stage is a gradual process. However, the curve does not pass through origin point, it is interpreted that intraparticle diffusion is not rate-determined.

**Table 5:** Kinetic constants for the intraparticle diffusion model for the adsorption.

Dyes	Biochars	k_i1_(mg·g^−1^·min^1/2^)	c_1_(mg·g^−1^)	R^2^	k_i2_(mg·g^−1^·min^1/2^)	C_2_(mg·g^−1^)	R^2^
CR	VM300	0.1976	4.11	0.9327	0.0606	5.37	0.0333
	VM500	0.7765	4.03	0.7534	0.3257	6.05	0.8854
	VM700	1.9187	5.06	0.9942	0.0621	13.10	0.2251
MB	VM300	0.2325	19.59	0.8991	0.0975	19.94	0.5562
	VM500	0.7076	8.93	0.9721	0.3606	12.40	0.8791
	VM700	0.4752	8.64	0.9159	0.1705	11.07	0.9542

### Possible mechanisms for adsorbing these 2 dyes

**Fig 6:**
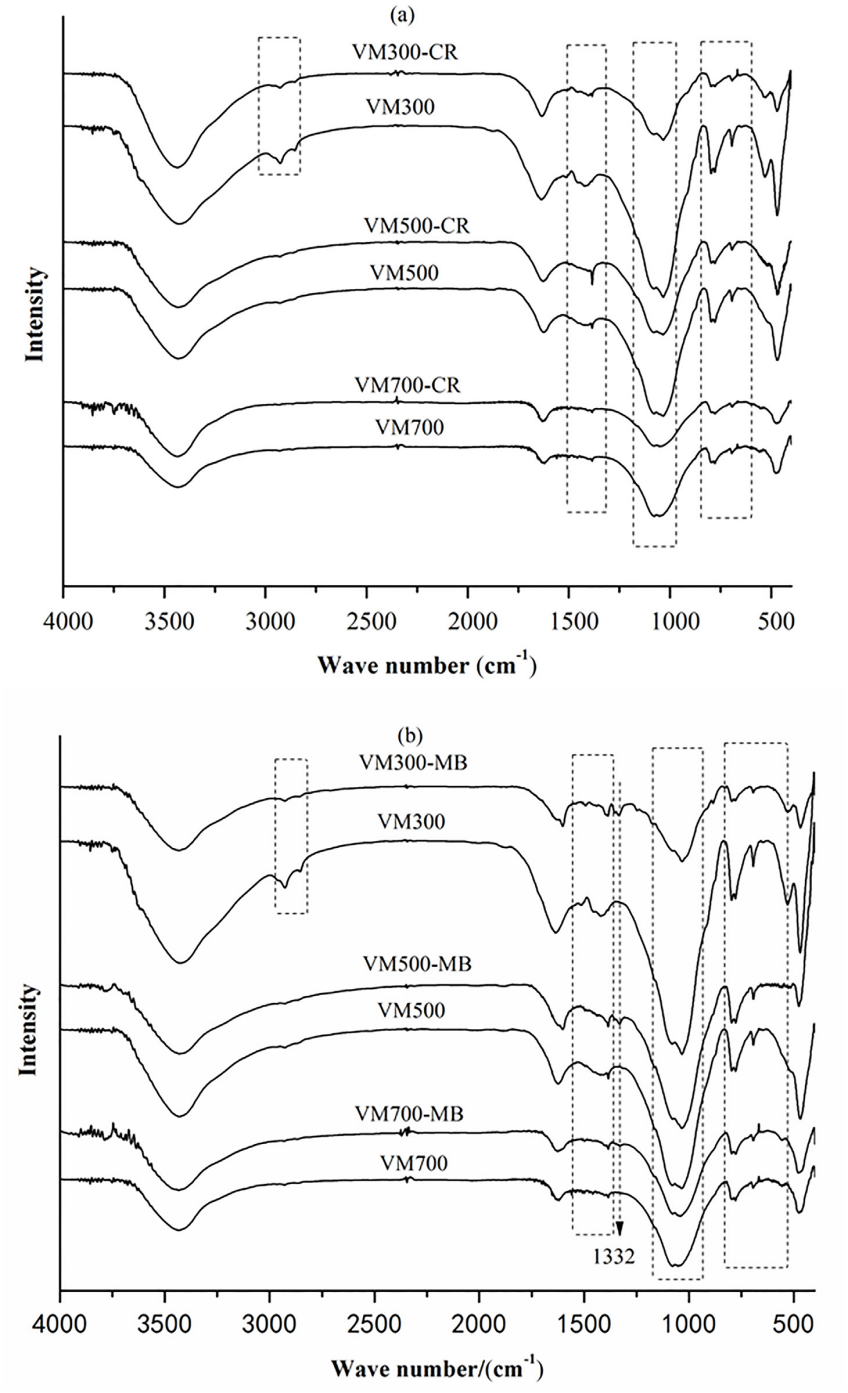
FT-IR spectras. (a) the biochars before (VM300, VM500 and VM700) and after (VM300-CR, VM700-CR and VM700-CR) the adsorption of CR; (b) the biochars before (VM300, VM500 and VM700) and after (VM300- MB, VM700- MB and VM700- MB) the adsorption of MB.

According to kinetics of the adsorption of these 2 dyes (see Fig 4), the adsorption can be rapidly equilibrated within 45 min, implying the chemical adsorption may be a dominant mechanism. In order to ascertain the chemical interaction between the dye molecules and the surface of the biochar, the FT-IR spectra of biochar and biochar-dyes are analyzed. As shown in Fig 6, the adsorption peaks of dye-bound biochar at 1515, 1451, 1421, 1385, 1034, and 800-500cm^−1^ display a reduced intensity of adsorption comparing to those of the dye-free biochar. Biochars retaining the MB dye show a new adsorption band at 1332 cm^−1^ (stretching vibration of methyl C–O, see Fig 6b) [41]. These indicate that the—NH_2_,—OH, carboxyl, carbonyl, and alkyl functional groups are involved in the possible chemical adsorption process. According to some referred work [42–44], the possible actions in the intensities of the adsorption bands mentioned above can be attributed to: 1) hydrogen bond formation between nitrogen and oxygen containing functional groups of dyes and biochars; 2) π-π dispersion interaction between the aromatic rings in the dyes and biochars; 3) the interaction of sharing electron, in which the carbonyl oxygen present in biochar can act as electron acceptor and the aromatic ring of CR or MB can be as an electron acceptor; 4) the electrostatic interaction between dyes and biochars, which can be proved by the investigated results in the effects of pH.

When the isothermal adsorption was investigated, the adsorption can be well described by the model of Langmuir. This result meant that a homogeneous surface containing equivalent sites are available for CR and MB adsorption. Moreover, a good fitness of Temkin for CR and MB adsorption also can be observed, suggesting the chemical adsorption was mainly attributed to electrostatic interactions [37].

**Fig 7:**
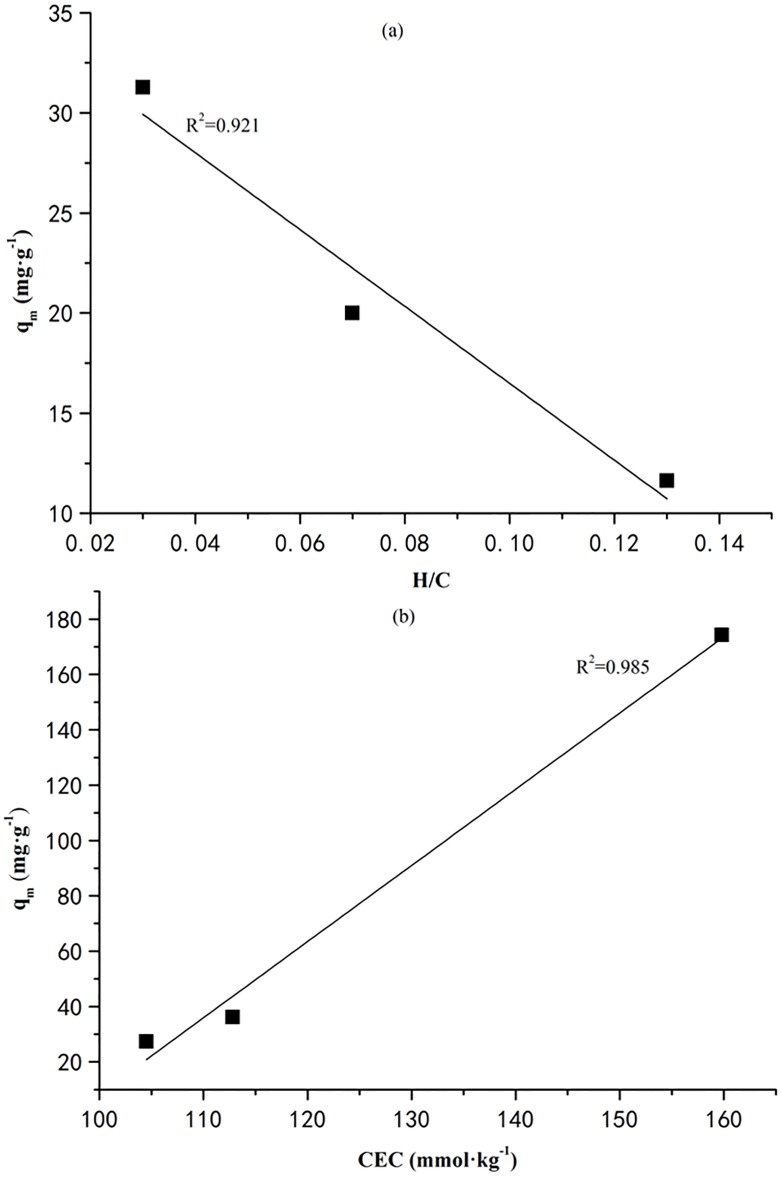
(a) The maximum amount of CR adsorption and the linear relationship with its aromaticity (H/C); (b) maximum adsorption capacity of biochar for retaining MB and its relationship with CEC of the biochar.

In addition, the pyrolysis temperature employed for producing vermicompost-derived biochar plays a great role in determining the amount of dye adsorbed by the biochar. In this work, biochar generated at a higher pyrolysis temperature retains a higher quantity of CR while retaining a relatively lower quantity of MB, which may be attributed to the changes of biochar natures. As stated above, electrostatic interactions could be the mainly mechanism for CR uptake by biochar. According to the characteristics of biochars listed in Table 1, the H/C decreased with increasing the pyrolysis temperature, indicating the aromaticity of biochar will be promoted. As a kind of electrostatic interaction, π-π dispersion interaction, which happened between the aromatic rings in the dyes and aromatic structure in graphite layer of biochars, will be strengthened consequently [43]. When the H/C and the maximum adsorption capacity of CR was correlated (see Fig 7a), the correlation coefficient (R^2^) was 0.921, again proving the π-π dispersion interaction controlled the CR adsorption. However, the similar relationship could not be observed on MB adsorption, suggesting different mechanism existed in MB adsorption.

According to the maximum adsorption capacity of MB by the biochar produced at different temperature, it could be found that the pyrolysis temperature negatively affected the MB uptake. As preliminarily analyzed in Table 1, the cation exchange may relate to the MB adsorption. Herein, the CEC of biochar at different pyrolysis temperature was correlated with the the maximum adsorption capacity of MB, and results was depicted in Fig 7b. The positive correlation could be observed with a relatively high correlation coefficient (R^2^) of 0.985. However, there was no similar relationship between CR adsorption and CEC of biochar. This results can prove that the cation exchange was the main mechanism for retaining MB using the biochar derived from vermicompost.

## Conclusions

Increasing pyrolysis temperature has a significant influence on the properties of biochar, which is consequently beneficial to CR adsorption while negative to MB uptake. The CR and MB adsorption are described better by the Langmuir and Temkin isotherm model rather than Freundlich model. The kinetic is well described by the pseudo-second-order model. The observed data suggest that the dosage of biochar has a significant impact on the adsorption of the dyes. The maximum adsorption by the biochar are 1.0g·L^−1^and 0.5g·L^−1^ for CR and MB, respectively. The promoted equilibrium pH exhibits negative to CR adsorption and positive to MB uptake. Electrostatic interaction of chemical adsorption is deduced as the dominant mechanism for both of CR and MB, in which the π-π dispersion interaction controls the CR adsorption, and the cation exchange is the main mechanism for retaining MB.

## Supporting Information

S1 Fig SEM images of biochars. (a) VM300, (b) VM500 and (c) VM700.
(TIF)

S2 Fig FTIR spectra of the biochars. (TIF)
