# Supplementary figures and images for: Removal of Congo Red and Methylene Blue from Aqueous Solutions by Vermicompost-Derived Biochars
Source: PLoS One. 2016 May 4;11(5):e0154562. doi: 10.1371/journal.pone.0154562 (PMC4856393; doi:10.1371/journal.pone.0154562)

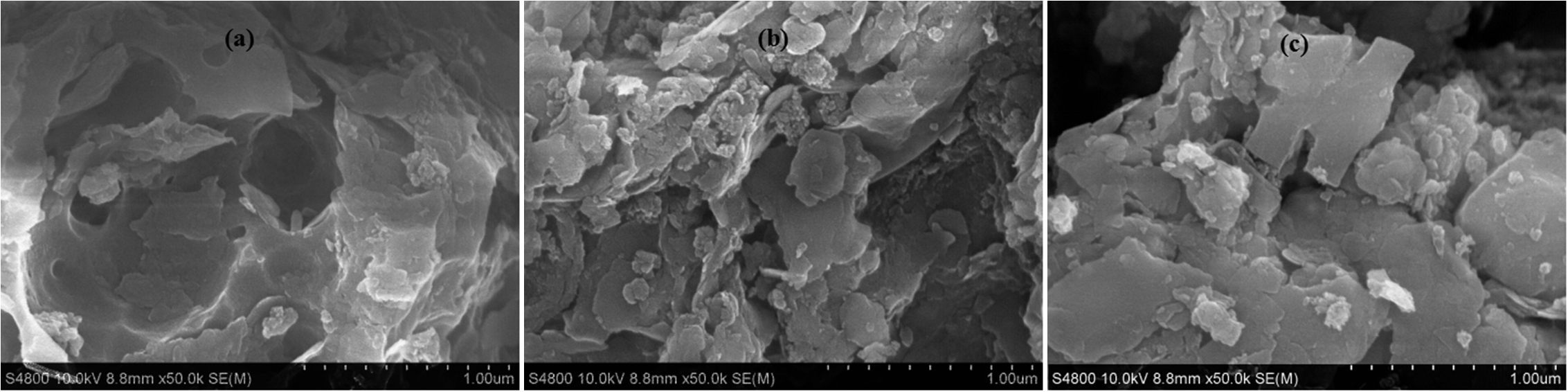

Supplement: S1 Fig — (a) VM300, (b) VM500 and (c) VM700. (TIF) [file pone.0154562.s001.tif]

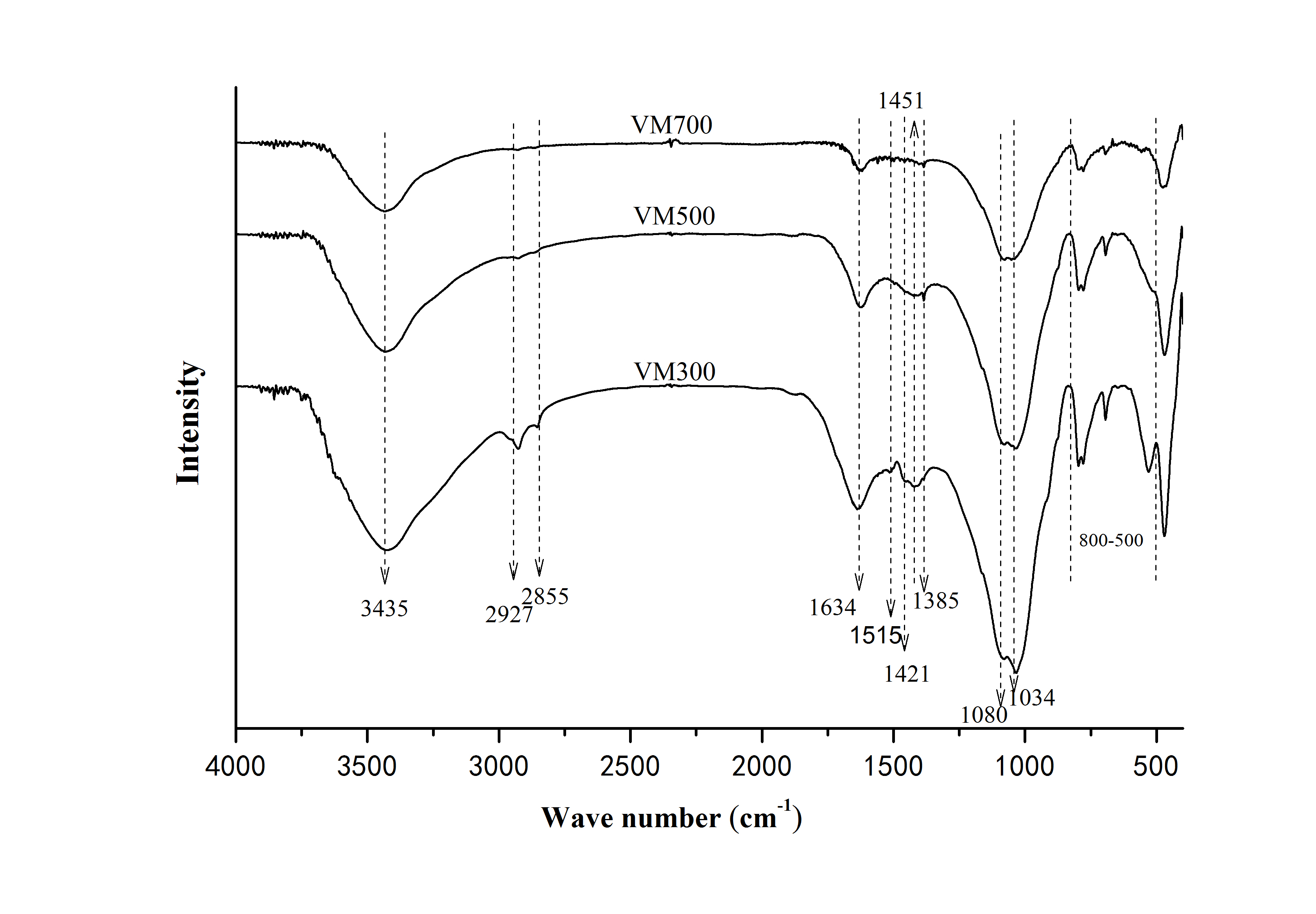

Supplement: S2 Fig — (TIF) [file pone.0154562.s002.tif]
